# In-Situ One-Step Direct Loading of Agents in Poly(acrylic acid) Coating Deposited by Aerosol-Assisted Open-Air Plasma

**DOI:** 10.3390/polym13121931

**Published:** 2021-06-10

**Authors:** Gabriel Morand, Pascale Chevallier, Cédric Guyon, Michael Tatoulian, Diego Mantovani

**Affiliations:** 1 Laboratory for Biomaterials and Bioengineering (CRC-I), Department of Min-Met-Mat Engineering and the CHU de Québec Research Center, Laval University, PLT-1745G, 2325 Rue de l’Université, Québec, QC G1V 0A6, Canada; gabriel.morand.1@ulaval.ca (G.M.); pascale.chevallier@crchudequebec.ulaval.ca (P.C.); 2 Laboratoire Procédés, Plasmas, Microsystèmes (2PM), Institut de Recherche de Chimie Paris (IRCP-UMR 8247), Chimie ParisTech-PSL, PSL Research University, 11 Rue Pierre et Marie Curie, F-75005 Paris, France; cedric.guyon@chimieparistech.psl.eu (C.G.); michael.tatoulian@chimieparistech.psl.eu (M.T.)

**Keywords:** aerosol-assisted deposition, open-air plasma, entrapment in coating, acrylic acid

## Abstract

In biomaterials and biotechnology, coatings loaded with bioactive agents are used to trigger biological responses by acting as drug release platforms and modulating surface properties. In this work, direct deposition of poly(acrylic acid) coatings containing various agents, such as dyes, fluorescent molecules, was achieved by aerosol-assisted open-air plasma. Using an original precursors injection strategy, an acrylic acid aerosol was loaded with an aqueous aerosol and deposited on silicon wafers. Results clearly showed that agents dissolved in the aqueous aerosol were successfully entrapped in the final coating. The effect of aerosols concentration, flow rate, and treatment time, on the coating morphology and the amount of entrapped agents, was also investigated. It was demonstrated that this process has the potential to entrap a tunable amount of any sensible water-soluble agent without altering its activity. To the best of our knowledge, this is the first time that the loading of an aqueous aerosol in coatings deposited by plasma from a liquid aerosol precursor is reported. This innovative approach complements plasma deposition of coatings loaded with bioactive agents from aqueous aerosols with the use of non-volatile liquid precursors.

## 1. Introduction

Coatings containing bioactive agents constitute one of the most successful value-added strategies for engineering material surfaces while preserving bulk properties. They are of special interest in the biomedical field, where specific interactions between biomaterials and the biological surrounding environment need to be promoted and triggered. In fact, these coatings can modulate surface properties while acting as drug delivery systems [1,2], for example, to confer antibacterial properties or cell-growth control [3,4,5]. In general, coatings loaded with bioactive agents are obtained by “wet” chemistry methods such as dip- or spray-coating [4,6]. However, such processes involve time-consuming steps and require large amounts of solvent thus increasing their cost and hindering their wide-scale production. Finally, they generally exhibit poor adhesion properties, which constitutes a major drawback for medical devices and implants [7].

In this context, atmospheric pressure plasma deposition (APPD) has emerged as an appealing approach for the manufacturing of coatings because it efficiently allows depositing a large variety of coatings from a limited amount of chemicals, with an even enhanced adhesion on a number of substrates. Furthermore, this process can be easily included in an open-air industrial production line, which makes it rapid and cost-efficient, if compared to low-pressure plasma [8,9]. However, when bioactive agents need to be loaded into the coating, their successful deposition induces non-trivial challenges. In fact, the sensitive bioactive agents (such as organic molecules, for example) are expected to be entrapped, homogeneously distributed, and unaltered by the reactive plasma environment.

In order to prevent bioactive agent denaturation, the coating deposition step is carried out under conditions known as soft-plasma polymerization, achieved by using dielectric-barrier discharge (DBD) sources [10,11]. In such an approach, the overall plasma reactivity is decreased to a point where it does not reasonably alter the structure of the molecules. However, soft-plasma polymerization is limited to the use of specific polymerizable precursors (PPs), essentially alkenes or siloxanes [12]. These PPs are injected in the discharge as a gas, as a vapor from the evaporation of volatile components, or as a liquid from nebulization of non-volatile components in the so-called aerosol-assisted APPD (AA-APPD). Since bioactive agents of greatest interest, such as proteins, antibiotics, or nanoparticles, are mostly non-volatile, they need to be dispersed into a solution and nebulized into the discharge.

Two strategies are generally adopted to disperse the bioactive agents into the solutions and inject them into the discharge. First, the bioactive agents are dissolved into the PP solutions, prior to nebulization [13,14]. However, this approach is restrictive as it requires the bioactive agents to be soluble, and stable in the PP solution [15]. To overcome this issue, a second strategy consists of dissolving the bioactive agents into water, which is then nebulized and injected in the discharge, while the PP is introduced separately as gas [16,17,18,19,20,21,22,23,24], or as vapor [25]. The mechanism associated with this approach has already been reported [24], and presents the main advantage of being appropriate for water-soluble agents [15]. Furthermore, this approach successfully allows the entrapment of several agents, including enzymes [17], proteins [16,18,19,20], and antibiotics [21,23]. Moreover, Lo Porto et al. evidenced that the bioactive agent release could be controlled by changing process parameters [23]. However, mainly gaseous precursors have been investigated, e.g., acetylene and ethylene gas or evaporated hexamethyldisiloxane [15], which strongly limit the choice of PP. Indeed, PPs in a liquid state such as acrylic acid [26], lactic acid [14,27], caprolactone-based molecules [10], methacrylate anhydride [11], ethylene glycol-based molecules [27,28], constitute promising candidates for the plasma-deposition of polymeric drug carriers and have never been investigated, to our best of knowledge [15].

**Figure 1:**
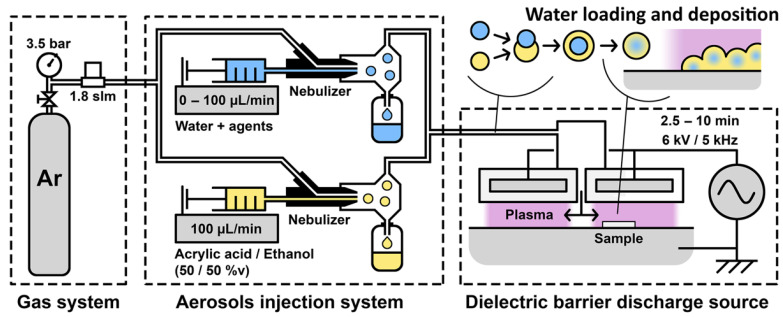
Scheme of the experimental set-up of the open-air aerosol-assisted atmospheric pressure plasma deposition; gas system feeds the aerosols injection system, aerosols are nebulized and mixed in the Ar flow, aerosols are injected into the dielectric barrier discharge to be deposited on silicon wafer samples. Scheme of the in-flight loading of the water droplets into the precursor aerosol, as depicted by the solvent exchange method, and deposition mechanism.

Therefore, in this work, a one-step deposition of coatings containing agents from a non-volatile liquid precursor, herein acrylic acid, using open-air AA-APPD was investigated. To do so, the liquid PP is simultaneously nebulized with water aerosol containing agents. This innovative strategy consists of the in-flight loading of water droplets inside hydrophilic acrylic acid ones, as displayed in Figure 1, and is based on the original solvent exchange method used in the field of aerosols [29,30]. The impacts of different parameters such as aerosols concentration, flow rate, and treatment time on the coating morphology and the amount of entrapped agents were evaluated, and discussed in accordance with the loading mechanism.

## 2. Materials and Methods

AA-APPD was performed with an AlmaPLUS reactor system (AlmaPlasma, Bologna, Italy). It consists of a gas system, a homemade aerosol injection system, and an open-air under-hood planar DBD source (Figure 1). The gas system was used to feed the aerosol injection system with a 1.8 standard liter per minute flow rate of Ar 99.999% (Messer Canada Inc., Mississauga, ON, Canada) by setting the pressure to 3.5 bar. The aerosol injection system consisted of two parallel nebulizers PEEK Mira Mist (Burgener, Mississauga, ON, Canada) allowing loading the Ar flow with a precursor aerosol and a water aerosol simultaneously. Nebulizers were fed by syringe pumps, which allowed controlling the aerosols flow rate. Both aerosols were mixed through 20 cm of gas line and a mixing chamber and then injected into the DBD. The DBD source consists of two high-tension electrodes placed above a grounded metallic table used as a sample holder. The inter-electrodes gap was set to 2 mm. High-tension electrodes are surrounded by 4.5 mm of dielectric high-density polyethylene. A 6 kV tension with a 5 kHz frequency was applied on the high-tension electrodes to produce the discharge. A 1 mm gap between the two high-tension electrodes was used to fill the DBD with the Ar flow loaded with the aerosols. AA-APPD was performed on 1×1 cm^2^ clean substrates cut from (100)-oriented silicon wafers with a thickness of 280 µm. The effect of deposition time was studied between 2.5 min and 10 min (Table 1). A 50/50% v mixture of acrylic acid 99% (Sigma-Aldrich, Hamilton, ON, Canada) and ethanol anhydrous (Commercial Alcohols, Brampton, ON, Canada) was used as precursor solution. Ethanol addition is expected to increase water aerosol loading into the precursor aerosol [31,32,33]. Ultrapure water from a Purelab Flex (Elga Veolia, Woodridge, IL, USA) was used as a water solution. The precursor flow rate was fixed to 100 µL/min while the effect of the water flow rate was studied between 0 µL/min and 100 µL/min (Table 1).

The morphology of the coatings was characterized with a scanning electron microscope (SEM) FEI Quanta 250 (FEI Company Inc. Thermo-Fisher Scientific, OR, USA) using a 7.5 kV acceleration voltage in secondary electron mode, at an observation distance of 10 mm. Prior to analyses, samples were coated with a thin gold-palladium film to obtain scanning electron images with improved quality. Coated samples were fractured in order to observe the cross-section of the coatings and measure their thickness. The coatings composition was investigated by an Attenuated Total Reflectance Fourier-Transform Infrared (ATR-FTIR) using a Cary 660 spectrometer (Agilent Technologies, Santa-Clara, CA, USA). In order to characterize the water aerosol deposition and its entrapment in the coating, the water solution was loaded with various tracers. Coatings were then deposited under condition C (Table 1) and characterized using three different characterization methods depending on the tracer. First, the water solution was loaded with commercial red food colorant (1 drop/mL, Club House, London, ON, Canada) and the resulting coating was observed with an optical microscope Olympus BX41M (Olympus America Corp., Center Valley, PA, USA) in bright field mode. Then, the water solution was loaded with CuSO_4_ (1 mmol/mL, Sigma-Aldrich, Hamilton, ON, Canada) and the resulting coating was observed with an energy dispersive X-ray spectrometer (EDX) EDAX (Ametek Material Analysis, Mahwah, NJ, USA) coupled with SEM. Finally, the water solution was loaded with fluorescent Lucifer Yellow CH, Lithium Salt (LY, 428 nm_ex_/536 nm_em_, 0.5 mg/mL, Thermo-Fisher Scientific, Waltham, MA, USA), and the resulting coating was observed with a confocal microscope LSM800 Axio Observer 7 (Carl Zeiss, Jena, Germany) using the z-stack mode. A total of 28 slices were recorded over a thickness of 7.29 µm using a 488 nm laser source and a 450–700 nm range of detection. Ultimately, quantification of deposited LY was performed. After overnight aging under ambient conditions, coatings were immersed in 500 µL of ultrapure water for 60 min, until complete dispersion and dissolution. The immersion solution was collected and placed in 96 multi-well plates and the fluorescence was recorded by means of a SpectraMax i3x Multi-Mode Plate Reader (Molecular Devices, San Jose, CA, USA). Quantification errors were extracted from uncertainties on the calibration curve plotted using 20 measurements from 0 µg/mL to 0.6 µg/mL in ultrapure water, and from the standard deviation calculated from five different coated samples. The various conditions under which AA-APPD was performed are reported in Table 1. The impacts of water flow rate (Table 1, conditions A, B, C, and D), deposition time (Table 1, conditions E, C, and F), and LY concentration (Table 1, conditions G, C, and H) were studied.

## 3. Results and Discussion

### 3.1. Poly(acrylic acid) Coating Deposition

**Figure 2:**
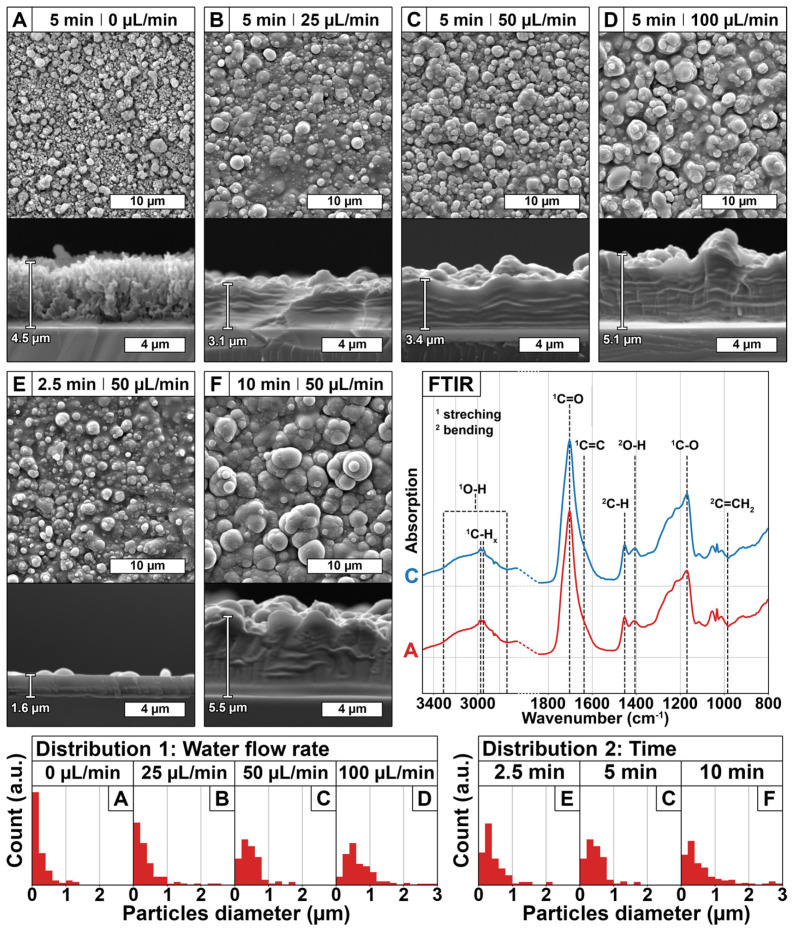
Scanning electron microscopy images of coatings surface morphology (×5000) and cross-section (×10,000) deposited with different water flow rate: 0 µL/min (**A**), 25 µL/min (**B**), 50 µL/min (**C**), and 100 µL/min (**D**); and for different time: 2.5 min (**E**), 5 min (**C**), and 10 min (**F**). Influence of plasma parameters, water flow (Distribution 1) and deposition time (Distribution 2), on the distribution of particles diameter, done on 200 particles. Fourier-transform infrared spectra of coatings chemistry deposited without water flow rate (FTIR, A, red spectrum), and with 50 µL/min water flow rate (FTIR, C, blue spectrum), and identification of major bonds signal.

Coatings were deposited under the conditions A to F listed in Table 1. Images of the surface and cross-section of coatings were obtained from SEM and are shown in Figure 2. The as-deposited coatings are composed of agglomerated particles, whose sizes and size distributions vary depending on the deposition conditions, as shown in Figure 2.

The coating deposition from acrylic acid, as a non-volatile precursor, was performed without water flow (condition A) and is further used as control. This coating appears to be porous, with a thickness of ~4.5 µm and composed of particles whose diameters are mostly smaller than 200 nm (Figure 2A). The addition of a flow rate of 25 µL/min of water during the plasma deposition process (condition B), leads to a different coating morphology and thickness, as clearly observed in Figure 2B. Indeed, the coating appears to be continuous whereas it was porous in condition A and its thickness decreases to ~3.1 µm from ~4.5 µm without water. This coating is also composed of particles that exhibit higher diameters than the ones previously obtained without water, as seen with the widening of the distribution until 1 µm, the decrease of the proportion of particles below 200 nm, and the apparition of particles larger than 2 µm (Figure 2B). This increase in the dimension of the particles is correlated to the broadening of the size of the droplets in the aerosol. In fact, due to the increased probability of in-flight collisions induced by the injection of the water droplets, the coalescence of the droplets, and in consequence their size, is increased. Moreover, in the presence of water, the shape of the particles appears more defined and more spherical, which is explained by increased surface tension of the droplets due to their coalescence. In fact, the water surface tension is higher than both acrylic acid and ethanol (71 mN/m against 28 mN/m and 21 mN/m, respectively at 30 °C according to suppliers). Regarding the coatings differences in terms of morphology and thickness in presence of water (Figure 2B) or not (Figure 2A), they can be related to the low vapor pressure of water compared to the one of ethanol (2.3 kPa against 5.8 kPa at 20 °C according to suppliers). In fact, a part of water remains liquid inside the particles after deposition, and this residual water then induces the solvation of the surrounding poly(acrylic acid) chains, their reorganization, and finally the loss of the porous structure, thus leading to a continuous and thinner coating. All these observations corroborate the mechanism of in-flight water loading in the precursor droplets, as depicted in Figure 1, and its successful deposition.

Then, the impact of some parameters as water flow rate and deposition time on poly(acrylic acid) coatings morphology and thickness was investigated. The water flow rate was increased progressively from 25 µL/min (Figure 2B) to 50 µL/min (Figure 2C) and 100 µL/min (Figure 2D), while keeping the deposition time constant (5 min). Whatever the water flow rate, the coatings remained continuous and included particles. Nonetheless, SEM analyses clearly evidence that incrementing the water flow rate from 25 µL/min to 100 µL/min increases the thickness of the coating from ~3.1 µm to ~5.1 µm, respectively. In the same manner, particle sizes are impacted, as their diameters increase, as seen by a progressive decrease of the proportion of particles below 200 nm and the apparition of particles larger than 3 µm (Figure 2—Distribution 1). Both effects were correlated to a higher number of water droplets in the gas flow due to the higher water flow, which increases the probability of in-flight collision between droplets, thus enhancing the coalescence of the droplets. Hence, the diameter of droplets increases, and so does acrylic acid deposition. Therefore, increasing the water flow rate increases the deposition rate.

Regarding the impact of treatment time on coatings thickness, while keeping the water flow rate constant at 50 µL/min, SEM images clearly evidence that increasing deposition time increases the thickness of the coatings: from ~1.6 µm for 2.5 min (Figure 2E) to ~3.1 µm for 5 min (Figure 2C), until ~5.5 µm for 10 min (Figure 2F). From these measurements, it appears that the coating thickness increases linearly with deposition time (R^2^ = 0.995), leading to a deposition rate value of ~0.6 µm/min (from 2.5 min to 10 min). Moreover, longer deposition time leads to an increase of the diameter of particles, as evidenced in SEM images as well as a change in the particles diameter distributions (Figure 2—Distribution 2): a decrease of the proportion of particles between 200 and 400 nm and an increase of the proportion of larger particles, up to 4 µm (Figure 2F). According to previous results, this is correlated to the enhanced coalescence of the droplets, meaning that the latter increases with deposition time.

After having demonstrated the impact of water presence, water flow rate, as well as deposition time on coatings morphology and thickness, and the composition of the coatings, were evaluated by FTIR analyses (Figure 2, FTIR). The spectra of coatings deposited without and with water, conditions A and C, respectively, are similar, meaning that the presence of water does not influence the plasma-induced process of acrylic acid polymerization. Moreover, these spectra display the characteristic bands of poly(acrylic acid) obtained by standard polymerization (“wet” chemistry), without any noticeable difference [34]. These main characteristic bands are the following: carboxylic acid O-H stretching at 2800 cm^−1^ to 3300 cm^−1^, C-H stretching at 2970 cm^−1^ and 2950 cm^−1^, C=O stretching at 1703 cm^−1^, H-C-H scissoring at 1452 cm^−1^, carboxylic acid O-H bending at 1406 cm^−1^, and carboxylic acid C-O bending at 1169 cm^−1^ [34]. Moreover, it should be emphasized that no residual traces of bands from the initial double bond carbon of the acrylic acid, C=C stretching at 1637 cm^−1^ and C=CH_2_ deformation out of the plane at 984 cm^−1^, are evidenced [34]. This observation allows us to conclude that the plasma polymerization occurs via the vinyl group and is indeed a soft-plasma polymerization process as expected with AA-APPD [12].

### 3.2. Agents Deposition and Entrapment in the Coating

**Figure 3:**
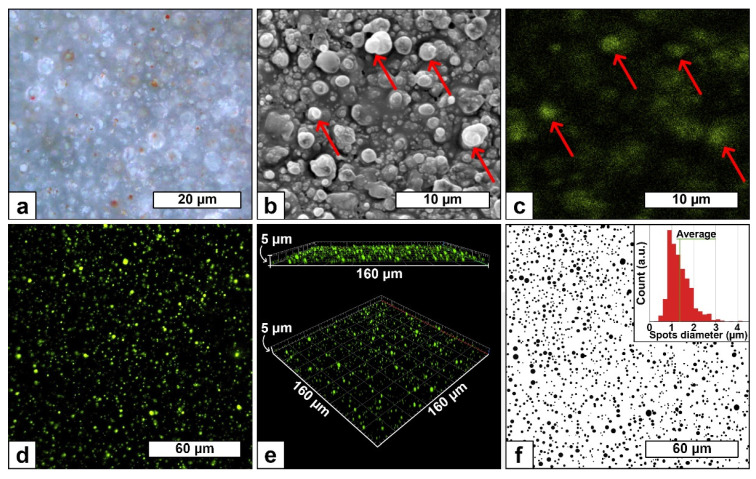
Characterization of different agents in coatings deposited with condition C (Water aerosol flow rate of 50 µL/min and deposition time of 5 min); bright field optical microscopy image (×100) of coating deposited with water loaded with 1 drop/mL of red colorant (**a**), scanning electron microscopy image of the surface morphology (×5000) (**b**) and corresponding energy-dispersive X-ray spectroscopy mapping of Cu (**c**) of coating deposited with water loaded with 1 mol/L of CuSO_4_, confocal microscopy image (×50, 428 nm_ex_/536 nm_em_) (**d**) and corresponding three-dimensional representations (**e**) of coating deposited with water loaded with 0.5 mg/mL of Lucifer Yellow, LY, and binarized image of the fluorescent spots of the confocal microscopy image “d” (**f**) and extracted spots diameter distribution on 1603 spots (f, graph, top-right).

The feasibility of depositing poly(acrylic acid) coatings using an original precursors injection strategy, liquid PP simultaneously nebulized with a water aerosol, while keeping its chemical composition, was demonstrated. In order to determine if the innovative strategy proposed herein has the potential to entrap agents in the coating, while keeping its molecular integrity, various tracers were dissolved in the water solution and deposited under condition C (Table 1). For this purpose, different agents were tested, from simpler to more complex ones: red food colorant, copper sulfate and Lucifer Yellow (fluorescent molecule). The resulting coatings are shown in Figure 3.

When loaded with red food colorant, red spots can be visualized in the image obtained by optical microscopy (Figure 3a). Their presence validates that the tracer, initially dissolved in the water solution, is indeed deposited. However, due to optical microscopy limitations, this result does not allow us to determine whether the red colorant is in fact entrapped in the coating or just sprayed. To assess the coating morphology while visualizing the loaded agent, CuSO_4_ was used. In fact, the resulting coating morphology and the Cu distribution within the coating can be analyzed simultaneously by SEM and by EDX mapping (Figure 3b,c, respectively). Foremost, the presence of an agent in the water solution does not seem to affect the coating morphology nor has an effect on the diameter of particles (Figure 3b) when compared to the coating without the agent dissolved in the water solution and deposited in the same condition (Figure 2C). Thanks to EDX mapping, the presence of Cu in the coating is evidenced. The Cu distribution appears homogeneous all over the coating, with some Cu-richer areas pointed out by red arrows in Figure 3c. By comparison with the corresponding SEM image (Figure 3b), the Cu-rich areas are corresponding to specific particles (Figure 3b, red arrows), suggesting that the Cu is mainly localized into these specific deposited particles. This result corroborates that the Cu is in the acrylic acid droplets during their polymerization and deposition, meaning that the Cu droplets are indeed loaded in the precursor aerosol, as foreseen. Therefore, the in-situ one-step direct loading of agents in poly(acrylic acid) coating deposited by aerosol-assisted open-air plasma is successful. However, this result just demonstrates that Cu is loaded in the last coating micrometer (SEM depth analysis) but not within all the coating thickness. Furthermore, the statement that the loaded agent will keep its molecular integrity has yet not been proven.

To do so, a sensitive fluorescent molecule, LY, was used, and the as-obtained coating was characterized by confocal microscopy. Moreover, this technique permits 3-dimensional imaging, which allows observation of the agent within all the coating thicknesses (Figure 3d,e). A homogeneous distribution of green and fluorescent spots, associated with the presence of LY, is observed in the 2D image (Figure 3d), meaning that the plasma process has not altered this sensitive molecule. In addition, 3D confocal microscopy images (Figure 3e) show that these fluorescent spots are well distributed within all the coating thicknesses. The round shape of fluorescent spots, whose diameters ranged between 800 nm–2 µm (Figure 3f—average spot size of 1.37 µm), corroborates that the LY is entrapped in acrylic acid droplets. If this value is compared with the particles diameter distribution extracted from the SEM image (Figure 2C), it corroborates the presence of LY in the largest droplets, as expected by the loading mechanism by coalescence.

### 3.3. Deposited Agent Quantification

**Figure 4:**
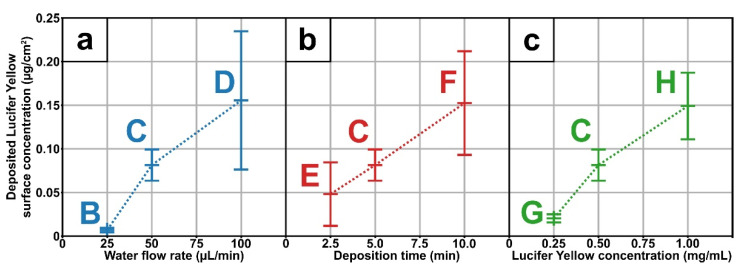
Quantification of Lucifer Yellow in coatings depos ited with different water flow rate (**a**): 25 µL/min (B), 50 µL/min (C), and 100 µL/min (D); for different time (**b**): 2.5 min (E), 5 min (C), and 10 min (F); and for different Lucifer Yellow concentration in the water aerosol (**c**): 0.25 mg/mL (G), 0.5 mg/mL (C), and 1 mg/mL (H).

This innovative approach was developed with the aim to efficiently load agents in poly(acrylic acid) coating thanks to AA-APPD, and to control its concentration for application as drug-release systems. That said, the first step was to quantify the amount of LY deposited and entrapped in the coatings depending on the deposition conditions, as reported in Table 1. Briefly, the influence of water flow rate (Figure 4a), deposition time (Figure 4b) and initial LY concentration (Figure 4c) on the LY loaded concentration inside the coating, in µg/cm^2^, was evaluated.

By increasing the water flow rate, the amount of deposited LY rises from 7 ng/cm^2^ ± 1 ng/cm^2^ for 25 µL/min (Figure 4a, B), to 81 ng/cm^2^ ± 17 ng/cm^2^ for 50 µL/min (Figure 4a, C), up to 155 ng/cm^2^ ± 78 ng/cm^2^ for 100 µL/min (Figure 4a, D). This result can be correlated to previous SEM images showing that increasing the water flow rate leads to higher particle diameters due to the enlargement of the size of the droplets formed in the aerosol. However, there is no linear correlation between the water flow rate and the deposited LY concentration (Figure 4a). Indeed, at 25 µL/min (Figure 4a, B), the LY concentration is quite low compared to 50 µL/min and 100 µL/min ones, as if a minimal water flow rate is needed to induce an effective loading of the agent during the coalescence of the droplets. Regarding the effect of deposition time, increasing it increases the amount of deposited LY from 43 ng/cm^2^ ± 35 ng/cm^2^ for 2.5 min (Figure 4b, E), to 81 ng/cm^2^ ± 17 ng/cm^2^ for 5 min (Figure 4b, C), and to 152 ng/cm^2^ ± 58 ng/cm^2^ for 10 min (Figure 4b, F). When correlated to SEM images, this LY concentration increase with longer deposition time can be expected, as the diameter of the deposited particles is larger, and the coating thickness is higher. Furthermore, the deposited LY concentration is linear with the coating deposition time (R^2^ = 0.998) in agreement with the constant deposition rate, as mentioned before (based on thickness evaluation from SEM images—Figure 2C,E,F). Finally, it can be observed that increasing water LY concentration increases the amount of deposited LY from 20 ng/cm^2^ ± 4 ng/cm^2^ for 0.25 mg/mL (Figure 4c, G), to 81 ng/cm^2^ ± 17 ng/cm^2^ for 0.50 mg/mL (Figure 4c, C), and to 149 ng/cm^2^ ± 37 ng/cm^2^ for 1.00 mg/mL (Figure 4c, H). Therefore, changing the initial LY concentration is one manner to control the amount of loaded agents without affecting others process parameters. This easy and promising approach will allow tuning released agent concentrations depending on the targeted drug delivery applications.

### 3.4. Loading and Deposition Mechanism

The parameters studied herein showed that it is possible to tune the coating morphology, thickness and concentration of the deposited agent. Furthermore, the results provide evidence that agent deposition is correlated to the amount of water inside the precursor aerosol. Indeed, an increase in water flow rate, i.e., t higher water concentration in the aerosol led to bigger poly(acrylic acid) particles (SEM images—Figure 2) and higher loaded agent concentration (Figure 4a). These observations mean that water is loaded in the precursor droplets prior to PP polymerization, as depicted in Figure 1. Moreover, this coalescence process allows maintaining the integrity of the agent of interest, as seen with the use of a fluorescent-sensitive molecule (Figure 3d–f). In addition, the spots size of the different tracers, investigated herein either red colorant (Figure 3a), CuSO_4_ (Figure 3c), and LY (Figure 3d), shows similar diameters, i.e., 2 µm and smaller. Moreover, coatings morphology seemed to be unmodified in presence of the agent (Figure 2C and Figure 3b). This suggests that the presence of the agent has little to no impact on the loading and deposition mechanism, meaning that this AA-APPD process would allow entrapping various water-soluble agents in poly(acrylic acid) coatings.

Finally, this study demonstrates that the coalescence of water and precursor droplets is a critical step for successful agent loading and deposition. Such loading arises from the decrease in surface tension of the droplets when water is loaded in the precursor droplets [29,30]. However, this mechanism suggests that entrapment will be only possible with hydrophilic precursor solutions. This would limit the choice of PPs to hydrophilic precursors. Nevertheless, ethanol addition into the precursor solution represents a promising method in order to increase its hydrophilic behavior and thus stimulates coalescence of water droplets with the aerosol of precursor, even though it is hydrophobic [31,32,33].

## 4. Conclusions

The developed one-step AA-APPD process, from a non-volatile liquid precursor, allowed depositing various water-soluble agents loaded in a poly(acrylic acid) coating. Fluorescent agent deposition showed that this process maintained agent integrity during deposition. Process parameters, including deposition time, water flow, and water agent concentration, allowed us to fine-tune both contents in loaded agents and coating morphology/thickness. A mechanism based on the coalescence of water and precursor droplets was proposed to explain the as-obtained coatings. Thanks to the versatility of this innovative procedure, the AA-APPD developed herein from a non-volatile liquid precursor would be transferable to any other liquid precursor of interest. By controlling the coating morphology and the loaded agent concentration, it could therefore be expected to control the agent release, making this approach appealing for designing specific drug-release systems.

## Figures and Tables

**Table 1:** Different conditions used during the aerosol-assisted atmospheric pressure plasma deposition by varying the water flow rate injected in the nebulizer, the deposition time and the Lucifer Yellow concentration in the water for the deposited agent quantification study.

Condition	Water Flow Rate (µL/min)	Deposition Time (min)	LY Concentration (µg/mL) ^1^
A	0	5	/
B	25	5	500
C	50	5	500
D	100	5	500
E	50	2.5	500
F	50	10	500
G	50	5	250
H	50	5	1000

^1^ Abbreviation: LY, Lucifer Yellow.

## Data Availability

The data presented in this study are available on request from the corresponding author.
